# Comparative Transcriptome Analysis of Short Fiber Mutants Ligon-Lintless 1 And 2 Reveals Common Mechanisms Pertinent to Fiber Elongation in Cotton (*Gossypium hirsutum* L.)

**DOI:** 10.1371/journal.pone.0095554

**Published:** 2014-04-18

**Authors:** Matthew K. Gilbert, Hee Jin Kim, Yuhong Tang, Marina Naoumkina, David D. Fang

**Affiliations:** 1 Cotton Fiber Bioscience Research Unit, USDA-ARS, Southern Regional Research Center, New Orleans, Louisiana, United States of America; 2 The Samuel Roberts Noble Foundation, Genomics Core Facility, Ardmore, Oklahoma, United States of America; New Mexico State University, United States of America

## Abstract

Understanding the molecular processes affecting cotton (*Gossypium hirsutum*) fiber development is important for developing tools aimed at improving fiber quality. Short fiber cotton mutants Ligon-lintless 1 (*Li_1_*) and Ligon-lintless 2 (*Li_2_*) are naturally occurring, monogenic mutations residing on different chromosomes. Both mutations cause early cessation in fiber elongation. These two mutants serve as excellent model systems to elucidate molecular mechanisms relevant to fiber length development. Previous studies of these mutants using transcriptome analysis by our laboratory and others had been limited by the fact that very large numbers of genes showed altered expression patterns in the mutants, making a targeted analysis difficult or impossible. In this research, a comparative microarray analysis was conducted using these two short fiber mutants and their near isogenic wild type (WT) grown under both field and greenhouse environments in order to identify key genes or metabolic pathways common to fiber elongation. Analyses of three transcriptome profiles obtained from different growth conditions and mutant types showed that most differentially expressed genes (DEGs) were affected by growth conditions. Under field conditions, short fiber mutants commanded higher expression of genes related to energy production, manifested by the increasing of mitochondrial electron transport activity or responding to reactive oxygen species when compared to the WT. Eighty-eight DEGs were identified to have altered expression patterns common to both short fiber mutants regardless of growth conditions. Enrichment, pathway and expression analyses suggested that these 88 genes were likely involved in fiber elongation without being affected by growth conditions.

## Introduction

Cotton fibers are single-celled trichomes that initiate from the ovule epidermal cells on or about the day of anthesis (DOA) [1]. Approximately 25% of the ovule epidermal cells differentiate into fiber cells during the initiation stage of cotton fiber development and subsequently undergo a period of rapid elongation known as the elongation stage [2], [3]. The rate of fiber elongation peaks at approximately 6 to 12 days post-anthesis (DPA) and nears cessation around 22 DPA [4]. During peak elongation fiber cells can increase in length at rates of 2 mm/day or more depending on environmental factors and genotypes [5]–[7]. Beginning at 12–16 DPA and overlapping with the elongation phase is the secondary cell wall (SCW) biosynthesis stage. During this stage cellulose is synthesized and deposited between the primary cell wall and the plasmalemma [8], [9]. Elongation and SCW biosynthesis continue until the fibers reach full length [25–35 mm in Upland cotton (*Gossypium hirsutum* L.) cultivars] [10], after which the cotton bolls open and the fibers desiccate under exposure to the environment. The environmental and genetic factors that influence the timing of these processes are shown to influence the development of desirable fiber traits such as lint yield and fiber quality [5], [11]–[13].

**Figure 1:**
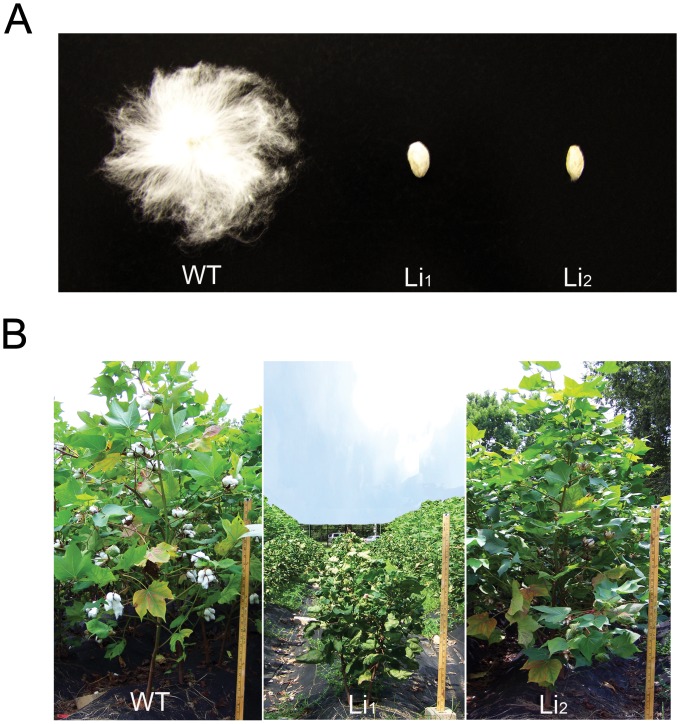
Cotton seed fibers (A) and plants (B) of wildtype DP5690 (WT), *Li_1_* mutant and *Li_2_* mutant. Plants were grown in the USDA-ARS Southern Regional Research Center field in New Orleans, LA.

Understanding the molecular mechanisms of fiber development is essential for cotton researchers to devise strategies for developing cotton lines with superior fiber quality. Furthermore, it is important to identify key genes that could be genetically engineered to improve fiber properties. Toward these goals, scientists have been using fiber mutants to study the molecular and genetic mechanisms of fiber development [14]–[17]. Among them, two short fiber mutants Ligon-lintless 1 and Ligon-lintless 2 (*Li_1_* and *Li_2_*, respectively) were extensively studied by our group [18]–[20] and others [14], [21], [22] in order to develop a comprehensive understanding of the molecular and metabolomic mechanisms related to cotton fiber length development. In a near-isogenic state with the cotton cultivars Texas Marker-1 (TM-1) or DP5690, both the *Li_1_* and *Li_2_* mutants have seed fibers that are extremely short (<6 mm) as compared to wild type (WT) fibers that are typically longer than 25 mm in length [18], [19], [23]–[25]. As a monogenic dominant trait, the short fiber phenotypes of *Li_1_* and *Li_2_* are similar (Fig. 1A). However, unlike the *Li*_2_ mutant, which appears morphologically similar to the WT plants with the exception of short seed fibers, the *Li_1_* mutant exhibits pleiotropy in the form of severely stunted and deformed plants in both the homozygous dominant and heterozygous state (Fig. 1B) [18], [23]. Although the *Li_1_* and *Li_2_* mutants have similar phenotype in cotton fiber, these two genes reside on different chromosomes with *Li_1_* on chromosome (Chr.) 22 and *Li_2_* on Chr.18 [18], [19], [26], [27]. These two mutants, when taken in combination, provide an excellent experimental system to find both common and mutant locus-specific mechanisms related to fiber elongation.

Analyzing the microarray or RNA-seq data collected to date for the *Li_1_* or *Li_2_* mutant is limited in one aspect by the fact that very large number of genes showed altered expression patterns between a mutant and its WT near isogenic line (NIL). For example, previous microarray data obtained from fibers of *Li_1_* or *Li_2_* showed approximately 1,500 to 2,500 differentially expressed genes (DEGs) including many genes that may be affected or regulated by environments [18], [19], [21]. It is difficult to decipher which of the genes are truly vital and common to fiber elongation-related processes, and which are due to different environmental, genetic and physiological cues if using only one mutant in an experiment as reported in all the previous studies. In recognition of this issue, we conducted the present experiment with two mutant lines and two growth conditions in order to identify the genes that are differentially regulated in both mutants, with the goal of identifying the common molecular mechanisms involved in cotton fiber length development. First we took advantages of our unique NILs of the *Li_1_* and *Li_2_* mutants. As reported earlier [18], [19], both *Li_1_* and *Li_2_* NILs were developed using the Upland cotton cultivar DP5690 as the recurrent parent. The *Li_1,_ Li_2_* and WT DP5690 are mutually near isogenic. Second, we conducted experiments in both field and greenhouse for *Li_2_* mutant, allowing the identification of genes impacted by environmental conditions and response to stress in this mutant. Third, we did a comparative analysis of transcriptome profiles between *Li_1,_ Li_2_* and WT to identify genes that had altered expression patterns in a short fiber mutant, regardless of the growth conditions (field or greenhouse) or the nature of the mutation (*Li_1_* or *Li_2_*). Our major objective was to identify common genes (or molecular mechanisms) that were essential to the fiber elongation regardless of environment or a specific mutation. Herein we report our findings.

## Materials and Methods

### Plant Materials

The *Li_1,_ Li_2_* and WT DP5690 used in the present study were mutual NILs. Development of these NILs was described in our earlier reports [18], [19]. For the greenhouse-grown *Li_2_* plants utilized in this study, growth and sample conditions were described in Hinchliffe et al. (2011) [19], and the growing period was between October, 2009 and March, 2010. Each plant was grown in an 18.9 L pot. A commercial service provider periodically sprayed pesticides to control insects or diseases. Automatic drip irrigation was used throughout the growing season. For field grown plants, a total of 200 *Li_1_*, 100 *Li_2_,* and 100 WT DP5690 plants were grown in a field at the USDA-ARS Southern Regional Research Center, New Orleans, LA in the summer of 2012. The distance between two plants within a plot was 30 cm. The plot distance was 45 cm. The soil type in this field was aquent dredged over alluvium in an elevated location to provide adequate drainage. Throughout the growing season, no pest control spray was applied. Supplemental sprinkle irrigation was provided when needed. Flowers were tagged and cotton boll sample collections were made before 10:00 a.m. and immediately placed on ice. To minimize environmental effects, boll samples were not collected from plants on the perimeter of the field and samples were only collected when 15–30 bolls were available for analysis. All samples of the same developmental stage were tagged and collected on the same day. Bolls were randomly separated into 3 replicates with 5–10 bolls per replicate. Bolls were then dissected, frozen in liquid nitrogen and stored at −80°C until further processing.

### RNA Isolation from Cotton Fibers

RNA was isolated as previously described [19]. To separate the fibers from the ovules the samples were shaken vigorously enough to break fibers without damaging the ovules. Isolation of RNA was conducted using the Sigma Spectrum Plant Total RNA Kit (Sigma-Aldrich, St. Louis, MO) with on-column DNaseI digestion and used according to the manufacturer’s instructions. RNA quantity was determined by using a Nanodrop 2000 spectrophotometer (NanoDrop Technologies Inc., Wilmington, DE). RNA integrity number (RIN) was determined for each sample using an Agilent Bioanalyzer 2100 and the RNA 6000 Nano Kit Chip (Agilent Technologies Inc., Santa Clara, CA). Only samples with RIN values of 7.0 or higher were used for expression analysis.

### Reverse Transcription Quantitative Real-time PCR (RT-qPCR)

The experimental procedures and data analysis related to RT-qPCR were performed according to the Minimum Information for Publication of Quantitative Real-Time PCR Experiments (MIQE) guidelines [28]. We used RNA samples from 12 DPA fibers in two biological replicates for cDNA synthesis and in two technical replicates for qPCR. The cDNA synthesis reactions were performed using the iScript cDNA Synthesis Kit (Bio-Rad Laboratories, Hercules, CA) according to the manufacturer’s instructions with 1 µg of total RNA per reaction used as template. The RT-qPCR reactions were performed with iTaq SYBR Green Supermix (Bio-Rad Laboratories) in a Bio-Rad CFX96 real time PCR detection system. The detail description of amplification parameters and calculation reported before [19]. Normalization of RT-qPCR data was performed by geometric averaging three internal control genes, including 18 S rRNA, ubiquitin-conjugating protein, and α-tubulin 4 [29]. The primer sequences of the 14 probe sets and the three internal control genes are listed in Table S1.

### Microarray Hybridizations and Data Analysis

The microarray technology used for this study was the commercially available Affymetrix GeneChip Cotton Genome Microarray (Affymetrix Inc., Santa Clara CA), comprised of 239,777 probes representing 21,854 cotton transcripts from a variety of expressed sequence tag (EST) databases. The source material for the EST data was derived from *G. arboreum*, *G. barbadense, G. hirsutum*, and *G. raimondii*. Labeling of the RNA was conducted using the Affymetrix GeneChip 3′ IVT Express Kit and cotton genome hybridizations were conducted according to the manufacturer’s protocols. Our earlier studies [18]–[20] indicated that a significant difference in both transcript profiles and fiber length measurement was observed at 12 DPA (peak elongation) between the *Li_1_,* or *Li_2_* and its WT NIL. Thus in this experiment, we used RNA samples from 12 DPA fibers (in two biological replicates) for microarray analysis. Probes sets demonstrating a two-fold or greater difference in expression levels between experimental samples were considered differentially regulated. Data normalization and the determination of statistically relevant deviations in expression patterns was performed as described [30].

### Gene Annotation Analysis

Microarray data obtained from greenhouse-grown *Li_2_* plants, and field-grown *Li_1_* and *Li_2_* plants were first subjected to Venn analysis utilizing BioVenn [31] to determine which probes demonstrate consistent expression profiles between experimental sets. To assist in the identification of biological processes represented in the data, Gene-Ontology Enrichment Analysis (GOEA) was performed using the agriGO Singular Enrichment Analysis (SEA) [32] by comparing to the *Gossypium raimondii* reference genome sequence [33]. The statistical test method used was the Fisher’s Exact test (significance level 0.05). Annotation of the probes was accomplished with Blast2Go [34]. For pathway analysis, MapMan software [35] was used to identify and illustrate pathways of interest using the January 12, 2013 *Gossypium hirsutum* mapping file. Co-expression analysis was conducted utilizing ATTEDII version 7.1 [36] with a mutual rank value of <200.

## Results and Discussion

### Effects of Growing Conditions (Field and Greenhouse) on Gene Expression

**Figure 2:**
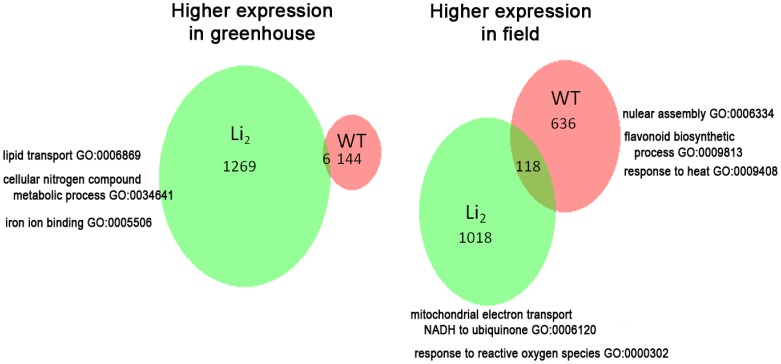
Probes showing altered regulation between greenhouse and field grown cotton. Each number represents the number of probes that showed altered expression between field and greenhouse conditions. Samples shown are 12 days post anthesis fibers. The included Gene Ontology labels are selected representative categories identified by Gene Ontology Enrichement Analysis conducted on AgriGO.

The data obtained in these experiments allowed for an analysis of the environmental effects on transcriptome data, i.e.; a field *vs* greenhouse comparison. This was useful for our purpose as it allowed the identification of transcripts affected by variable environmental conditions, which could then be excluded from consideration of strictly fiber-related transcripts. Although it is known that many fiber-related genes are environmentally impacted [5], the exclusion of these genes permitted a more targeted analysis of the genes that are essential to fiber elongation. It also allowed us to investigate how the mutant line differed from its WT in responding to environmental stressors, providing insight into the interaction between stress response and fiber elongation. Microarray analysis was conducted on WT and *Li_2_* 12 DPA bolls collected from cotton plants grown in both field and greenhouse conditions. This comparison identified 150 probes in the WT that were expressed higher in the greenhouse than in a field, and 754 probes that were expressed higher when grown in field conditions (numbers are sums shown in orange ovals of Fig. 2). Gene enrichment analysis of the probes higher in the field showed that genes related to nucleosome assembly (GO:0006334), flavonoid biosynthetic process (GO:0009813), and response to heat (GO:0009408) were enriched in the field conditions (Fig. 2 and Table S2). Probes showing higher expression patterns in the greenhouse in WT were not enriched in any particular GO category to a statistically significant degree due to smaller number of probes, however did consist of ethylene and ap2 erf domain-containing transcription factors, expansin, and probes related to NADH dehydrogenase. The results of a similar analysis conducted in the *Li_2_* mutant differed dramatically from what was observed in the WT, with 1,275 probes showing higher expression in the greenhouse and 1,136 probes showing higher expression in the field (numbers are sums shown in green ovals of Fig. 2). The probes showing higher expression in *Li_2_* in the greenhouse were enriched in lipid transport (GO:0006869), cellular nitrogen compound metabolic process (GO:0034641), and iron ion binding proteins (GO: 0005506), whereas, probes showing higher expression in the field were enriched in mitochondrial electron transport (GO:0006120) and response to reactive oxygen species (GO: 000302) (Fig. 2 and Table S2). Only 124 probes showed the same expression profile in WT and *Li_2_* in both field and greenhouse conditions. This difference between WT and *Li_2_* in response to the environmental conditions is profound, as very few probes showed similar expression profiles between the two genotypes, (i.e., only 6 common probes were higher in the green house and only 118 were higher in the field).

Gene enrichment analysis for *Li_2_* specific greenhouse/field condition response identified two categories of probes that are of particular interest; probes related to mitochondrial electron transport (GO:0006120) and response to reactive oxygen species (ROS) (GO:0000302) were both enriched in *Li_2_* in the field relative to their expression in the greenhouse. Probes for genes in these categories were not enriched in WT. It has been suggested in previous studies that the inability of certain *Gossypium* species and *G*. *hirsutum* mutants to produce long fibers may be due to the inability to modulate ROS homeostasis [19], [37], [38]. The mitchondrial electron transport gene NADH dehydrogenase (GhiAffx.21609.1.S1_at, GhiAffx.53261.1.A1_at, GhiAffx.45916.1.A1_s_at) and NADH plastiquinone reductase (GhiAffx.4260.1.S1_at, GhiAffx.61308.1.S1_at, GhiAffx.9732.1.A1_at) were among those highly expressed in *Li_2_* in the field condition. Because the field conditions presented both abiotic and biotic stresses that were absent or minimized in greenhouse conditions, it was likely that the field grown *Li_2_* plants needed to divert limited cellular resources to manage these additional stresses, leading to higher ROS accumulation and an even higher expression of ROS homestasis genes.

**Figure 3:**
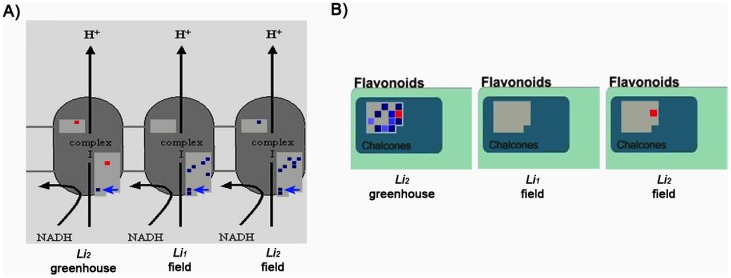
MapMan software illustrates different stress response of the short fiber mutants. Probes showing differential regulation were analyzed by MapMan software. The identification of processes affected differently in the wildtype and mutant lines was done manually. A) The *Li_1_* and *Li_2_* mutants showed increased (blue) expression of NADH dehydrogenase in field conditions as compared with greenhouse. Blue arrow indicating the common probe GhiAffix45916.1. B) *Li_2_* in greenhouse conditions exhibited increase chalcone synthase-related expression, which were not replicated in field conditions.

Expression levels of mitochondrial-related genes were studied across all three microarray data sets. In field conditions *Li_1_* had 7 probes (Ghi.7225.1.s1_s_at, Ghiaffx.53261.1.a1_at, Ghi.7032.2.a1_at, Ghi.7032.2.s1_s_at, Ghiaffx.21609.1.s1_at, Ghiaffx.18012.1.s1_at, Ghiaffx.45916.1.a1_s_at), and *Li_2_* had 9 probes (Ghi.7032.2.s1_s_at, Ghi.7032.2.a1_at, Ghiaffx.3647.1.s1_at, Ghiaffx.5964.1.s1_s_at, Ghi.7225.1.s1_s_at, Ghiaffx.53261.1.a1_at, Ghiaffx.18012.1.s1_at, Ghiaffx.45916.1.a1_s_at, Ghi.648.1.a1_at) up-regulated that had high sequence identity to NADH dehydrogenases, whereas in the greenhouse *Li_2_* had only one probe up-regulated, Ghiaffx.45916.1.a1_s_at (blue squares in Fig. 3A). The 2 probes down-regulated (red squares in Fig. 3A) consisted of Gra.1550.1, which has sequence identity to a Choline transporter-related transcript (AT4G38640) and Ghi.648.1.a1_at, which has sequence similarity to an NADH dehydrogenase. The probe that was up-regulated in all 3 data sets in the *Li* mutants is homologous to the probe GhiAffx.45916.1, a subunit of NADP dehydrogenase (NAD2) (shown by a blue arrow in Fig. 3A). These results further demonstrated the potential relevance of ROS and stress response in fiber developing processes.

Chalcone synthase is the upstream enzyme in the flavanoids synthesis pathway responsible for production of secondary metabolites (flavonols, proanthocyanins and anthocyanin) that are often produced in response to stresses [39], [40]. Here, the chalcone synthase-related probes in WT plants had significantly higher expression in field conditions than in the greenhouse as indicated by a significant enrichment in flavonoid biosynthetic processes (GO:0009813) (Table S2). Additionally, pathway analysis utilizing MapMan software indicated that under greenhouse condition, *Li_2_* had higher expression levels of chalcone synthase than WT (Fig. 3B). Thus, all of the plants in the field (WT, *Li_1_* and *Li_2_*), and the *Li_2_* plants in the greenhouse had high levels of genes related to flavonol production relative to the WT plants in the greenhouse. This further supports the hypothesis that the *Li_2_* mutant, even in greenhouse conditions, were in a stressed state. Ghi.6103.1 that codes for *chalcone synthase 3* (GhCHS3) (Fig. 3B, red squares) was the only identified flavonoid probe that decreased in *Li_2_* in both field and greenhouse conditions. The remaining probes that increased under field conditions demonstrate varying degrees of homology to Transparent Testa 4 (TT4) (8 probes) and TT5 (2 probes), both naringenin-chalcone synthases.

In brief, it was likely that field-grown plants were under more stressful conditions than greenhouse grown plants. Short fiber mutants (more specifically *Li_2_* mutant) commanded higher expression of genes related to energy production and transport such as mitochondrial electron transport or responding to ROS in order to fight against the stresses than the WT.

### Identification and Annotation of DEGs Common to both Short Fiber Mutants Regardless of Growth Conditions

**Figure 4:**
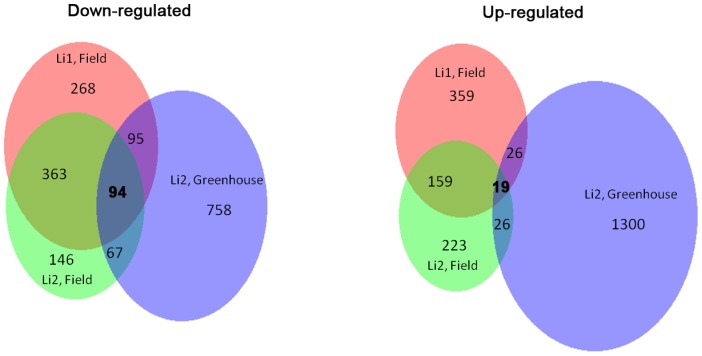
Venn Diagrams illustrating the similarities and differences between the experimental groups. Each number represents the number of probes showing different expression levels (>2 fold difference) between the mutants and the wildtype near-isogenic line.

Although the *Li_1_* and *Li_2_* mutations are caused by different genes located on different chromosomes, both result in a similar short fiber phenotype. Thus it is possible that probes which are similarly altered in expression pattern between a mutant and WT in both field and greenhouse conditions are highly likely to be fiber elongation-related or specific genes. In all three data sets, 113 probes are commonly affected in the mutants in comparison with WT with 94 down-regulated and 19 up-regulated (Fig. 4). Of these 113 probes that showed altered regulation, 25 also showed to be differentially regulated between field and greenhouse, implying their altered regulation could be an environmentally controlled factor and were excluded from further consideration. The remaining 88 probes are likely the genes specific to fiber elongation. Among them, 66 genes were annotated by blastn or blastx and were used for further analyses (Table 1 for annotated genes and Table S3 for full list).

**Table 1:** Annotation of common DEGs identified from both *Li_1_* and *Li_2_* mutants regardless of growth conditions.

No.	Probes	HomologousGene	G raimondiilocus
**1**	Ghi.7820.1.S1_s_at	18S ribosomal RNA (mitochondria)	Gorai.013G213000
**2**	GraAffx.32667.2.A1_s_at	18S rRNA (mitochondrial)	Gorai.013G213000
**3**	Ghi.581.1.S1_at	2-oxoglutarate (2OG) and Fe(II)-dependent oxygenase	Gorai.002G036400
**4**	GhiAffx.6286.1.S1_at	4-coumarate:CoA ligase (4CL)	Gorai.009G005900
**5**	Gra.1375.1.A1_at	actin 5 (ACT5)	Gorai.013G022400
**6**	GhiAffx.33535.1.S1_at	actin depolymerizing factor 5 (ADF5)	Gorai.008G035300
**7**	Ghi.1209.1.S1_at	ARM repeat superfamily protein	Gorai.010G025700
**8**	Ghi.8448.1.S1_x_at	beta-tubulin 1 (BTub1)	Gorai.004G211800
**9**	GraAffx.8388.1.S1_s_at	cellulose synthase-like protein	Gorai.009G066500
**10**	Ghi.3452.1.S1_s_at	cellulose synthase-like protein	Gorai.009G222300
**11**	GhiAffx.23257.1.S1_s_at	cellulose synthase-like protein	Gorai.009G066500
**12**	Ghi.2235.1.A1_s_at	chaperonin-60kD, ch60	Gorai.007G151700
**13**	Ghi.1908.1.S1_s_at	cofactor assembly	Gorai.007G003100
**14**	Ghi.8534.1.A1_s_at	cyclin-U2-1	Gorai.004G164400
**15**	Ghi.2840.3.S1_s_at	cysteine proteinase	Gorai.013G224100
**16**	GhiAffx.39816.1.S1_s_at	cytidine deaminase 1	Gorai.013G228000
**17**	GhiAffx.39795.1.S1_at	EF hand calcium-binding protein family	Gorai.008G075000
**18**	Ghi.68.1.A1_s_at	fasciclin-like arabinogalactan protein 1	Gorai.008G155400
**19**	Ghi.5801.1.A1_at	GDSL esterase/lipase	Gorai.011G103900
**20**	Ghi.5444.1.S1_at	gibberellin 20-oxidase	Gorai.004G149700
**21**	GhiAffx.11707.1.A1_at	gland development related protein 23-like	Gorai.004G208300
**22**	GhiAffx.8010.1.S1_at	glycolipid transfer protein	Gorai.005G138500
**23**	GhiAffx.19944.1.S1_at	glycoprotein membrane precursor GPI-anchored	Gorai.005G041200
**24**	Ghi.5081.1.S1_s_at	glyoxal oxidase-related protein	Gorai.002G125000
**25**	Ghi.632.1.S1_at	GroES-like zinc-binding alcohol dehydrogenase family protein	Gorai.002G066100
**26**	Gra.2833.1.S1_at	homeodomain-leucine zipper protein 56 (HDL56)	Gorai.003G041500
**27**	Ghi.5889.1.A1_x_at	HXXXD-type acyl-transferase family protein	Gorai.012G006700
**28**	Ghi.5889.2.S1_s_at	HXXXD-type acyl-transferase family protein	Gorai.012G006600
**29**	Ghi.7819.1.A1_at	hydrolase, alpha/beta fold family protein	Gorai.011G272800
**30**	Ghi.5515.1.A1_s_at	iron-binding protein (Fer1)	Gorai.006G184700
**31**	GraAffx.1241.1.S1_s_at	leucine-rich receptor-like protein kinase (LRPKm1)	Gorai.009G166500
**32**	Ghi.6301.1.S1_s_at	lung seven transmembrane receptor family protein	Gorai.007G019200
**33**	Ghi.6548.1.S1_s_at	MAP kinase-like protein	Gorai.002G096100
**34**	GhiAffx.29423.1.S1_s_at	MAR-binding protein	Gorai.004G245100
**35**	Ghi.5146.1.A1_x_at	NADP-dependent malic enzyme	Gorai.009G048600
**36**	GhiAffx.6438.1.S1_at	nodulin family protein	Gorai.007G034700
**37**	GhiAffx.21685.1.S1_at	nuclear transport factor 2 (NTF2)	Gorai.013G010700
**38**	Ghi.7430.2.S1_s_at	octicosapeptide/Phox/Bem1p (PB1) domain-containingprotein/tetratricopeptide repeat (TPR)-containing protein	Gorai.013G214100
**39**	Ghi.1352.1.S1_s_at	O-fucosyltransferase family protein isoform 1	Gorai.003G038000
**40**	Ghi.10656.1.S1_s_at	photosystem I subunit PsaD (PSAD)	Gorai.005G042000
**41**	GhiAffx.51155.1.S1_s_at	PIP protein (PIP2;7)	Gorai.011G098100
**42**	Ghi.5186.1.A1_at	plant invertase/pectin methylesterase inhibitor superfamily	Gorai.001G018200
**43**	GhiAffx.33585.1.S1_at	plant invertase/pectin methylesterase inhibitor superfamily	Gorai.002G031100
**44**	Ghi.8118.1.S1_at	putative carboxyl-terminal proteinase	Gorai.005G180300
**45**	GraAffx.34131.2.S1_x_at	pyridoxal phosphate phosphatase (PHOSPHO2)	Gorai.011G067300
**46**	Ghi.4013.2.S1_at	root iron transporter protein IRT1	Gorai.011G049700
**47**	GhiAffx.22857.1.A1_at	rps16 (chloroplast)	Gorai.001G180700
**48**	GhiAffx.43008.1.S1_at	SAUR family protein	Gorai.001G017600
**49**	GhiAffx.24789.1.S1_at	SAUR family protein (SAUR54)	Gorai.N011800.1
**50**	Ghi.5484.1.S1_s_at	SKU5-like 5 protein	Gorai.009G189900
**51**	Ghi.978.1.S1_at	tetratricopeptide repeat-like superfamily protein isoform 1	Gorai.013G142800
**52**	GhiAffx.44664.1.S1_at	thiosulfate sulfurtransferase	Gorai.007G049000
**53**	GhiAffx.53295.1.A1_at	UDP-glucuronosyl and UDP-glucosyl transferase	Gorai.008G273200
**54**	Ghi.9654.1.S1_s_at	UDP-glycosyltransferase UGT73C14	Gorai.008G273200
**55**	Ghi.3235.1.A1_at	UDP-glycosyltransferase UGT73C14	Gorai.009G411800
**56**	Ghi.10822.1.S1_at	xyloglucan endotransglucosylase/hydrolase (XTH2)	Gorai.003G033600
**57**	Ghi.4013.1.A1_at	zinc transporter 10 precursor	Gorai.011G049700
**58**	Ghi.10311.1.S1_s_at	SNARE protein Syntaxin 1 and related proteins	Gorai.006G148600
**59**	Ghi.4533.1.A1_x_at	SNARE protein Syntaxin 1 and related proteins	Gorai.006G148600
**60**	GhiAffx.7289.1.S1_at	SNARE protein Syntaxin 1 and related proteins	Gorai.006G148600
**61**	Ghi.7279.1.S1_at	ABC transporter	Gorai.009G022400
**62**	GhiAffx.58403.1.S1_at	G1/S-specific Cyclin D	Gorai.005G185600
**63**	GhiAffx.23478.1.S1_at	NTKL-BINDING PROTEIN 1	Gorai.001G120300
**64**	GhiAffx.4465.1.S1_s_at	F-BOX/LEUCINE RICH REPEAT PROTEIN	Gorai.009G049800
**65**	GhiAffx.44162.1.S1_s_at	Extracellular protein with conserved cysteines	Gorai.007G359700
**66**	Ghi.8451.1.S1_s_at	integral to membrane (GO:0016021)	Gorai.001G148000

Among the fiber elongation specific genes, actin depolymerizing factor (ADF5) (GhiAffx.33535.1) was decreased in all conditions analyzed. ADF family proteins have previously been shown to affect the 3-dimensional structure of actin filaments [41] and to alter the disassociation rates of actin subunits [42]. The down-regulation of GhADF1 in transgenic cotton show altered fiber length and strength [43] and GhADF5 has been shown to localize to elongating cells of the root stem in *A. thaliana* [44]. As shown in Table 1, actin (Gra.1375.1.A1_at) and tubulin (Ghi.8448.1.S1_x_at) were commonly down-regulated in short fiber mutants. It was proposed that actin and microtubule play important roles in fiber elongation [45]. Functional analysis showed that actin was indeed required for fiber elongation [46].

Two probes (Ghi.3235.1 and Ghi.9654.1.) showing altered regulation in all conditions are different regions of the same gene, UGT73C14, which codes for an UDP glycosyltransferase that glycosylates ABA *in vivo* and *in vitro* [47]. One probe (GhiAffx.53295.1.A1_at) also codes for UDP-glucuronosyl and UDP-glucosyl transferase, but has not been characterized in the context of elongation and warrant further investigation. Ghi.6548.1.S1 codes for a MAP kinase-like protein that was previously identified as preferentially expressed from elongating fibers in *G. hirsutum* by subtractive PCR but remains otherwise uncharacterized [48]. Two probes, GhiAffx.43008.1 and GhiAffx.24789.1 code for SAUR (Small Auxin Up RNA) family genes, which comprise the largest family of auxin-responsive genes. However, some members have also reportedly been associated with cell expansion [49]. This is potentially evidence of a substantial and specific link between auxin regulation and fiber elongation.

Two probes that decreased in all conditions analyzed code for either plant invertase or pectin methylesterase (PME) inhibitors (GhiAffx.33585.1 and Ghi.5186.1). Invertases catalyze the hydrolysis of sucrose, and PMEs inhibit the enzyme that catalyzes the de-methylesterification of pectins, a process important in initiation and for the polysaccharides role in the primary cell wall of cotton fibers [50], [51]. *Li_1_* and *Li_2_* have previously demonstrated a decrease in de-esterified pectin localized to the primary cell wall during elongation stages as indicated by antibody staining [52]. Previous studies have also demonstrated that there are at least 5 PMEs showing fluctuating expression profiles throughout fiber initiation, elongation, and transition to secondary cell wall synthesis, indicating that different PME regulated different fiber development processes [53]. It was further demonstrated that the timing of individual PME gene expression differed between the longer fibered Pima (*G. barbadense*) and Upland (*G. hirsutum*) species in such a manner that suggested the timing of PME activity affected the onset of the transition stage from elongation to secondary cell wall synthesis, in turn affecting length of the fiber [53]. Since it is suggested that PME activity generally corresponds to longer fibers [54]–[56], it is possible that the increased expression of PME inhibitor(s) identified in our study play a role in inhibiting mid or late elongation stage PMEs, thus low levels of inhibitor would induce early transition to secondary cell wall synthesis. This would effectively decrease the elongation period. In support of this, the PME inhibitor probed by Ghi.5186.1 also exhibited decrease expression ratios of 0.42 in *Li_1_* and 0.58 in *Li_2_* at 3 DPA (data not shown).

### Gene Ontology Analysis of the DEGs Common to both Short Fiber Mutants

**Figure 5:**
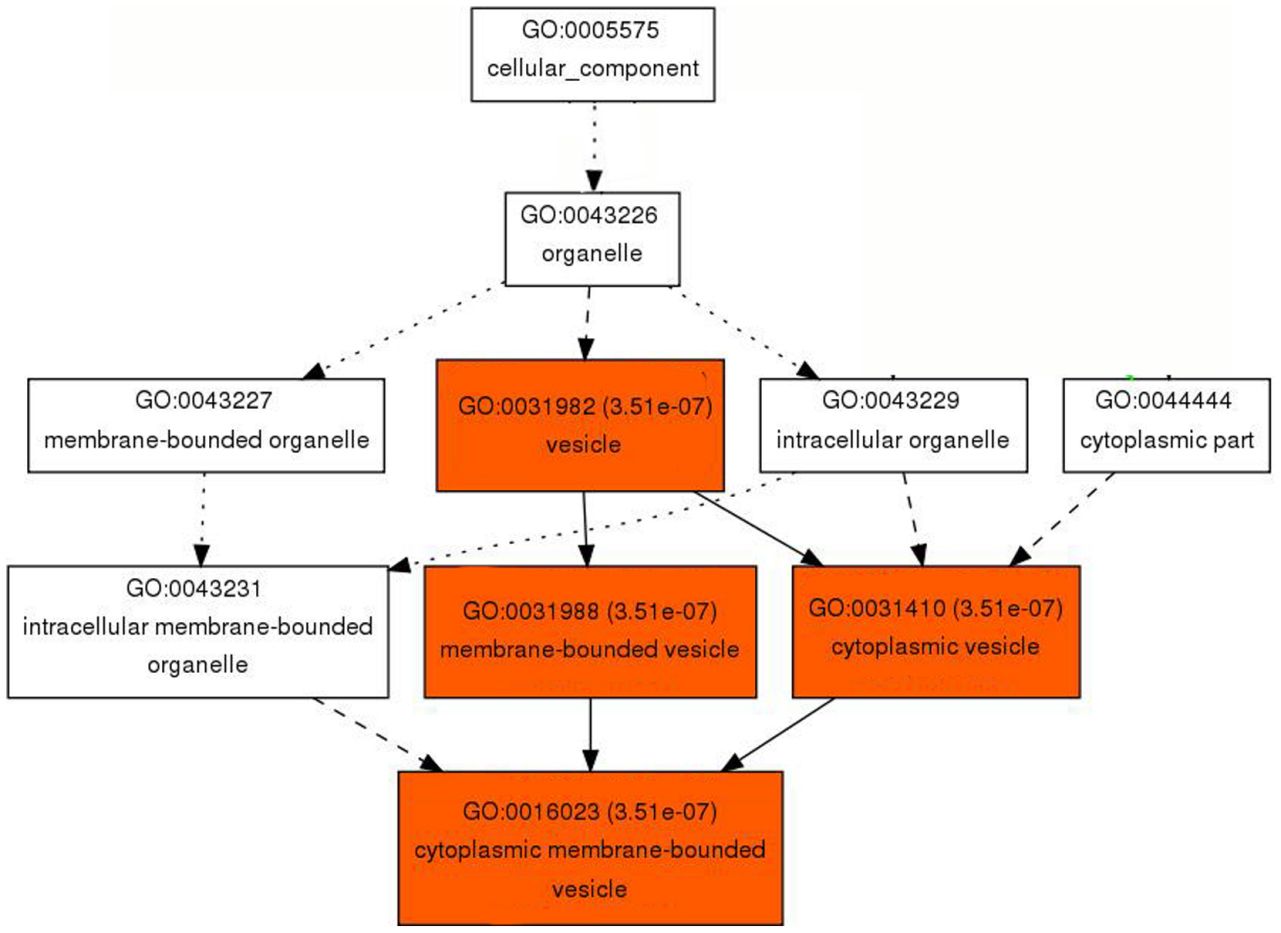
Identification of a potential cellular component in *Li_1_* and *Li_2_* mutants. AgirGo SEA analysis identified that nine of the DEGs presented in Table 1 were involved in vesicle transportation. The significant terms (adjusted *P*≤0.05) were shown in red color boxes, whereas non-significant terms were shown as white boxes. Solid, dashed, and dotted lines represented two, one and zero enriched terms at both ends connected by the line, respectively.

To identify the potential biological processes governing differential expressions of genes that affect fiber length development of the two short fiber mutants, we analyzed the identified 88 DEGs using agriGo SEA analyses. The GO enrichment analysis classified 9 DEGs as a group that is involved in vesicle transportation (GO:0031982) in cotton fiber cells (Fig. 5). The vesicle plays an important role of carrying membrane components to the growing site of elongating cells [57], [58]. Among the 9 vesicle-related DEGs (Table 2), fasciclin-like arabinogalactan protein (*GhFLA1*, Ghi.68.1.A1_s_at) was reported as an essential cotton fiber gene for fiber elongation and primary cell wall biosynthesis [59].

**Table 2:** Common DEGs involved in vesicle (GO:0031982) in both *Li_1_* and *Li_2_* mutants regardless of growth conditions (P value, 1.7e-11; FDR, 3.1e-10).

DEGs	Annotation
Ghi.68.1.A1_s_at	fasciclin-like arabinogalactan protein 1
GhiAffx.19944.1.S1_at	glycoprotein membrane precursor GPI-anchored
GraAffx.1241.1.S1_s_at	leucine-rich receptor-like protein kinase
Ghi.5186.1.A1_at	plant invertase/pectin methylesterase inhibitor superfamily
Ghi.7819.1.A1_at	hydrolase, alpha/beta fold family protein
Ghi.8118.1.S1_at	putative carboxyl-terminal proteinase
Ghi.7402.1.S1_at	Protein of unknown function, DUF642
Ghi.8451.1.S1_s_at	integral to membrane
Ghi.2840.3.S1_s_at	cysteine proteinase

### Co-expression Analysis of Annotated DEGs

**Figure 6:**
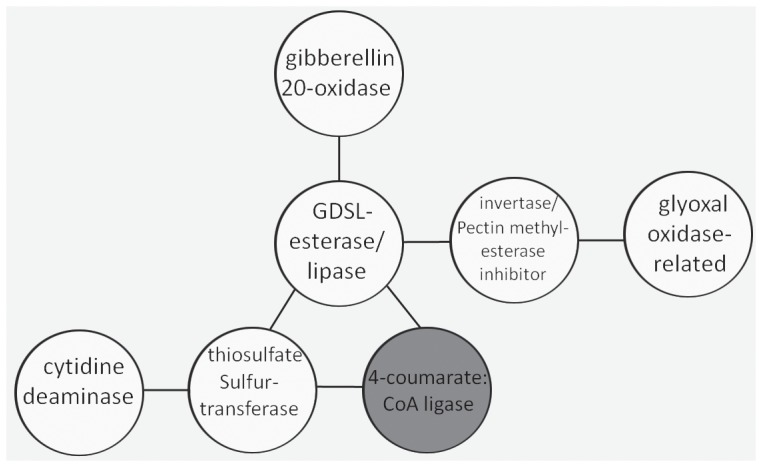
Co-Expression analysis. The ATTED-II database of genes co-expressed in Arabidopsis was utilized to identify potential interactions among the list of 66 targeted genes (see text). Circle with gray background indicates gene that is up-regulated, circles with white background indicate down regulated genes. A line connecting two genes means a mutual rank value of <200.

Beginning with our list of genes showing altered regulation in both mutants in all three data sets and no variability between field and greenhouse (Table 1), we wanted to identify genes that were previously identified as being co-expressed, and further support their roles in a common pathway and their involvement in the phenotype of the mutant plants. Subjecting the aforementioned list to the ATTED-II Arabidopsis co-expression database revealed a putative gibberellic acid-regulated pathway (Fig. 6). Several GDSL esterase/lipase family genes have been found with giberellin-responsive elements (P-box and GARE motif) in their 5′ upstream regulatory regions [60]. These genes are characterized by the presence of a conserved motif and have been shown to be involved in a wide range of functions, including stress response and development [61], [62], however to our knowledge this is the first report of a possible link with fiber development. The only gene up-regulated in the identified pathway, 4-coumarate-coenzyme A ligase, has been shown to play a role in lignin deposition in the plant cell wall of several species, which has exhibited a decrease in plant height when the gene was suppressed [63], [64]. In cotton fibers, lignin is a structural component of the primary cell wall, and genes that are involved in lignin deposition are up regulated in parallel with fiber elongation [65]. Finally, an additional topic that is re-visited in this analysis is the role of ROS-related genes in fiber development identified by this and other studies. Experimental evidence has demonstrated that the membrane bound glyoxal oxidases, which decreases in the mutants examined here, are known to produce hydrogen peroxide [66], [67], which is then used as a cofactor for lignin-synthesizing peroxidases [68]. Thus, the genes represented in Fig. 5 could help explain the apparent perturbation to ROS-related processes affected in the *Li_1_* and *Li_2_* mutants.

### Cell-wall Precursors

**Figure 7:**
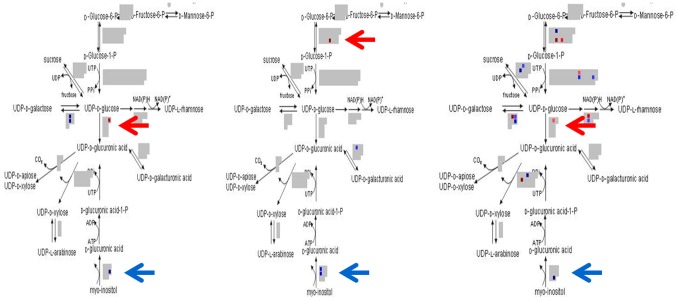
MapMan software illustrates the cell wall precursor pathway and effects of the *Li_1_* and *Li_2_* mutations. Enzymes involved in the synthesis of UDP-D-glucaronic acid are altered in both *Li_1_* and *Li_2_* mutants. Blue indicates up-regulated probes and red indicates down regulation. The arrows point to steps of the pathway that are discussed in more detail in the text.

Because our analysis was focused on cell wall development we utilized MapMan software to determine which steps in the synthesis of cell wall-related polysaccharides are affected in the mutants. Fig. 7 illustrates that the conversion of myo-inositol to D-glucaronic acid by myo-inositol oxygenase (MIOX), an early step in the synthesis UDP-D-glucaronic acid, was affected. Between the 3 experimental groups, there are 4 separate probes that were up-regulated (Fig. 7, blue arrows) (*Li_1_*/field, GraAffx.9655.1; *Li_2_*/Field, Ghi.8187.2, and GhiAffx.4265.1; *Li_2_*/greenhouse, Gra.2699.2). tBLASTx analysis of these EST sequences revealed GraAffx.9655.1 shares highest sequence identity with AtMIOX4, where as the other three share highest identity with AtMIOX1. Likely related to this, MapMan software also revealed the alternative pathway for UDP-D-glucaronic acid synthesis contains probes for genes that were down-regulated (red dots). The conversion of UDP-D-glucose to UDP-D-glucaronic acid is mediated by UDP-glucose 6 dehydrogenase, which was down-regulated in *Li_2_*/greenhouse (Gra.2095.2) and *Li_1_*/field (Ghi.8750.1) (red arrows). This gene was unaffected in *Li_2_*/field, however GhiAffx.64086.1, which codes for phosphofructokinase 3 and mediates the formation of D-Glucose-1-P, was down-regulated, likely indicating an alternative site of regulation (red arrow). The regulation of UDP-D-glucaronic acid is a vital step, as it serves as the common precursor for arabinose, xylose, galacturonic acid, and apiose residues in the cell wall. It has been reported in null Arabidopsis mutants that the limited supply of myo-inositol generally prevents the effective induction of this pathway as an alternative to UDP-glucose formation [69]. Thus it remains a strong possibility that the UDP-D-glucose levels and other polysaccharides necessary for cell wall development were perturbed and insufficient in the *Li_1_* and *Li_2_* mutant fiber tissues_._

### Verification of Microarray Results by RT-qPCR Analysis

To test the reliability of microarray data, RT-qPCR analysis was performed for a subset of 14 genes selected from Table S3 showing altered regulation in *Li_1_*, *Li_2_* (field) and *Li_2_* (greenhouse) as compared to the wild type. Expression of selected genes was tested at 12 DPA of fiber development, the common time point between greenhouse and field experiments. Overall, the results of RT-qPCR analysis were consistent with results of microarray for 14 selected genes (Table S4).

## Conclusions

The development of cotton fibers is a very complicated and poorly understood biological process, however understanding this process is vital for the targeting of genes to use in the creation of value-added crop. We have developed a genetic model system consisting of two short fiber cotton mutants, *Li_1_* and *Li_2_*, which when combined together with their near-isogenic WT line allows for the study of genes and processes specific to fiber elongation. Here we analyzed multiple transcriptome profiles obtained from *Li_1,_ Li_2_* short fiber mutants and their WT grown under different environmental conditions. We classified the differentially expressed genes into two groups: one was mainly affected by environmental conditions, and the other was largely regulated by *Li_1_* and *Li_2_*. Our results provide new insight to how environmental factors affect fiber elongation by transcriptional regulation. Further, the short list of 88 genes required for fiber elongation without being affected by environmental conditions would warrant further investigation in hope to identify targets for improving cotton fiber property.

## Supporting Information

Table S1 **Primer sequences used for RT-qPCR.**
(XLSX)

Table S2 **Complete results of gene ontology analysis.**
(XLSX)

Table S3 **Expression levels of probes showing altered regulation in** ***Li_1_, Li_2_*** **(field) and** ***Li_2_*** **(greenhouse) as compared to the wild type (WT).**
(XLSX)

Table S4 **Comparison between RT-qPCR and microarray under different growing environments.**
(XLSX)
